# Feelings of Contrast at Test Reduce False Memory in the Deese/Roediger-McDermott Paradigm

**DOI:** 10.3389/fpsyg.2021.686390

**Published:** 2021-09-13

**Authors:** Sara Cadavid, Maria Soledad Beato, Mar Suarez, Pedro B. Albuquerque

**Affiliations:** ^1^School of Medicine and Health Sciences, Universidad del Rosario, Bogotá, Colombia; ^2^Faculty of Psychology, University of Salamanca, Salamanca, Spain; ^3^School of Psychology, University of Minho, Braga, Portugal

**Keywords:** false memories, false recognition, DRM paradigm, disqualifying monitoring, memory error-editing processes, multiple critical lures per list, backward associative strength

## Abstract

False memories in the Deese/Roediger-McDermott (DRM) paradigm are explained in terms of the interplay between error-inflating and error-editing (e.g., monitoring) mechanisms. In this study, we focused on disqualifying monitoring, a decision process that helps to reject false memories through the recollection of collateral information (i.e., recall-to-reject strategies). Participants engage in recall-to-reject strategies using one or two metacognitive processes: (1) applying the logic of mutual exclusivity or (2) experiencing feelings of contrast between studied items and unstudied lures. We aimed to provide, for the first time in the DRM literature, evidence favorable to the existence of a recall-to-reject strategy based on the experience of feelings of contrast. One hundred and forty participants studied six-word DRM lists (e.g., spy, hell, fist, fight, abduction, mortal), simultaneously associated with three critical lures (e.g., WAR, BAD, FEAR). Lists differed in their ease to identify their critical lures (extremely low-BAS lists vs. high-BAS lists). At recognition test, participants saw either one or the three critical lures of the lists. Participants in the three-critical-lure condition were expected to increase their monitoring, as they would experience stronger feelings of contrast than the participants in the one-critical-lure condition. Results supported our hypothesis, showing lower false recognition in the three-critical-lure condition than in the one-critical-lure condition. Critically, in the three-critical-lure condition, participants reduced even more false memory when they could also resort to another monitoring strategy (i.e., identify-to-reject). These findings suggest that, in the DRM context, disqualifying monitoring could be guided by experiencing feelings of contrast between different types of words.

## Introduction

During the last decades, memory researchers have intensely explored the underlying mechanisms of memory distortions, and have shown a particular interest in false memories (Gallo, 2006, 2010). One of the most widely employed procedures to induce false memories in controlled settings is the Deese/Roediger-McDermott (DRM) paradigm (Deese, 1959; Roediger and McDermott, 1995). In this paradigm, participants study lists of words (e.g., sour, candy, sugar, etc.), all of them associated with a non-presented critical lure (e.g., SWEET). In a subsequent memory task, participants often claim to recall or recognize the critical lure (false memories) along with the studied items (true memories) (Roediger and McDermott, 1995). Numerous experimental manipulations have revealed that falsely remembered critical lures present highly compelling memorial evidence of the occurrence of the event (e.g., Beato et al., 2013; Boldini et al., 2013; Thakral et al., 2019; Brainerd et al., 2020; Howe and Akhtar, 2020; H. Liu et al., 2020; Beato and Arndt, 2021; Huff et al., 2021; Z. Liu et al., 2021; Wang et al., 2021).

In order to explain false memories, the two main theories are the fuzzy-trace theory or FTT (e.g., Brainerd and Reyna, 1990; Reyna and Brainerd, 1995; Brainerd et al., 2008) and the activation-monitoring framework or AMF (e.g., Roediger et al., 2001a,b). Despite differences between FTT and AMF, both agree to propose the interplay of two types of processes: error-inflating and error-editing.^1^ These processes would work together to increase true memories, but they would operate in opposite directions in false memory (Arndt and Gould, 2006). Thus, whereas error-inflating processes would increase the likelihood to produce false memories, error-editing processes would reduce it.

The main aim of this research was to study error-editing processes in associative false memories and, in particular, the monitoring process, which has been identified as key to reduce false memories (e.g., Roediger et al., 2001b; Gallo, 2004; Gallo et al., 2006; Gallo and Lampinen, 2016; Coane et al., 2020; Huff et al., 2020). The monitoring process can be generally described as a decision process that helps participants allocate the source of mentally activated information, eventually reducing false memory (e.g., Gray, 2016; Roediger et al., 2001b; cf. Jou et al., 2018, who proposed that the ability to establish an appropriate decision criterion to monitor is based on what the observer monitors against). This monitoring process can be classified into diagnostic and disqualifying monitoring (e.g., Gallo, 2004, 2006; Gallo et al., 2006; Gallo and Lampinen, 2016; Nieznański et al., 2018; Moore et al., 2020). This division is based on the decisional processes around the avoidance of false memory.

On the one hand, diagnostic monitoring relies on the expectations generated around the decision making and happens when the critical lure is rejected due to an absence of recollection. In those cases, the dubious event (i.e., the critical lure) is rejected following a reasoning such as “if I had studied that item (e.g., my favorite fruit), I would recall it; as I do not remember it, it must not have been presented” (Gallo and Lampinen, 2016). On the other hand, disqualifying monitoring involves deciding whether the questionable event (i.e., the critical lure) was studied or not is made based on the recollection of collateral evidence. In these cases, certain information is recalled, and the recollection of that memory eliminates the possible occurrence of the questionable event. The recollection of collateral information to reject a dubious event is called “recall-to-reject” (e.g., Gallo, 2004; Gallo and Lampinen, 2016; Moore et al., 2020). Research has suggested that recall-to-reject can work through two different metacognitive processes: (1) participants apply the logic of mutual exclusivity, or (2) participants experience a feeling of contrast between studied items and unstudied critical lures (Gallo and Lampinen, 2016).

Examples of how recall-to-reject can occur following a logic of mutual exclusivity come from studies in which DRM lists are very short (e.g., three items). In that case, participants could reject the critical lures at test by remembering all the studied words of each list (i.e., exhaustive recall/recognition), which leads to extremely low false recognition rates (Gallo, 2004). Another example of the use of the logic of mutual exclusivity to avoid false memories is the finding that highly identifiable critical lures are more likely to be rejected (Carneiro et al., 2009, 2012). In this case, participants would apply a particular type of logic of mutual exclusivity that has been called “identify-to-reject,” and that would follow such a reasoning: “I did not encode A because, first, I remember to notice that A was the theme of the list, and second, I realized that A was not presented”. In addition, as noted above, participants can also engage in a type of recall-to-reject based on a feeling of contrast between the studied items and the unstudied critical lures (Gallo and Lampinen, 2016; Moore et al., 2020). In these cases, a sort of automatic attributional process might be intervening to reject the false memory, which would follow a reasoning like this: “This word (i.e., A, the critical lure) seems familiar to me, but I probably have this feeling just because another related word was actually presented (i.e., B, a studied item), so I will reject A” (Brainerd et al., 2003; Lampinen et al., 2005). Evidence for this strategy came from a study in which participants rated words for pleasantness and then completed an old/new recognition test in which they also had to think out loud and say whatever came to their minds during the retrieval process (Lampinen et al., 2005). This study found that participants sometimes noticed differences between the recollection experience of studied and unstudied items (“Cup—is new. I don’t remember seeing cup, but I remember seeing mug”). These results suggest that participants sometimes experience a feeling of contrast between items when performing a recognition test. In this context, it is worth noting that, even though the theoretical explanation of the feeling-of-contrast strategy is strongly related to the DRM associative illusion, to date, there is no empirical evidence that supports the idea that participants engage in this type of monitoring process in the DRM paradigm. To fill this gap, we examined whether, in the DRM paradigm, participants apply a recall-to-reject strategy based on feelings of contrast between items. As far as we know, ours is the first attempt in the literature to tackle this particular question directly.

To test the existence of the feeling-of-contrast strategy, we need a procedure that precludes the possibility to apply the logic of mutual exclusivity and allows the feeling-of-contrast strategy. As mentioned above, there are two main examples of how recall-to-reject can occur following a logic of mutual exclusivity: exhaustive recall/recognition and identify-to-reject strategies. One could argue that, since DRM lists are usually long, participants cannot resort to exhaustive recall/recognition because it is tough to remember all the studied items. However, this argument is not sufficient to affirm that participants are not engaging a mutual exclusivity logic as we also need to consider the possibility that participants apply an identify-to-reject strategy. In this regard, it should be noted that a typical DRM list includes one critical lure and words with high backward associative strength (BAS, the association from studied items to the critical lure). When using DRM lists with these characteristics (i.e., high BAS and one critical lure), participants are prone to engage in an identify-to-reject strategy, a subtype of the logic of mutual exclusivity. Two mechanisms could trigger identify-to-reject in these lists. First, when there is only one critical lure per list, participants could identify this word as the theme of the list, they could be aware of the absence of this word in the study list, and, therefore, they might reject it at test (i.e., identify-to-reject strategy). Instead, we think that if the DRM lists included multiples critical lures, a condition used in the present study, it would be more difficult to engage in an identify-to-reject strategy. Second, in previous studies, a positive correlation has been found between BAS and identifiability indexes of the critical lures (e.g., Beato and Cadavid, 2016). That is, critical lures were more easily identifiable as the theme in high-BAS lists than in low-BAS lists. Therefore, participants are less likely to engage in an identify-to-reject strategy in DRM lists with lower backward associative strength.^2^

To prevent the use of mutual exclusivity logic (both via exhaustive recognition and identify-to-reject strategies) and analyze whether experiencing feelings of contrast could guide error-editing processes in DRM studies, we used DRM lists with multiple critical lures and extremely low levels of backward associative strength. Specifically, we manipulated two independent variables in this study: the number of critical lures per DRM list presented at test (one vs. three) and BAS (high vs. low). Including three actual critical lures per list at the recognition test would increase the likelihood that participants engage in a feeling-of-contrast strategy (i.e., more critical lures would increase the chances that participants have a feeling of contrast). Also, it seems likely that including all the three critical lures of our lists in the recognition test could diminish the probability of engaging in an identify-to-reject strategy (i.e., participants would not be able to explicitly identify the three critical lures of each list to reject them). Furthermore, to make it even less likely that participants engage in an identify-to-reject strategy, we included lists with the minimum possible BAS levels. These extremely low-BAS levels served as a proxy for low-identifiability levels. Therefore, lists with three critical lures and extremely low BAS would constitute the experimental condition in which we prevented the use of mutual exclusivity logic and, instead, foster a feeling-of-contrast strategy. Hereunder, these ideas are explained in more detail.

With respect to the number of critical lures per list, previous studies have reported that two- or three-critical-lure DRM lists produce robust false memories (e.g., Beato and Díez, 2011; Beato et al., 2012; Cadavid et al., 2012; Beato and Arndt, 2014, 2017; Beato and Cadavid, 2016; Cadavid and Beato, 2016, 2017; Arndt and Beato, 2017; Pitarque et al., 2018). In these previous studies, it is unlikely that participants would have used the logic of mutual exclusivity to reject the critical lures because they would not be able to (1) remember all the studied items or (2) explicitly identify the two or three themes of the list (i.e., critical lures) to reject them. In contrast, we expected that participants would engage more often in a feeling-of-contrast strategy following a reasoning like this: “This word (i.e., A, one of the critical lures) seems familiar to me, but I probably have this feeling just because another related word was actually presented (e.g., studied word B or critical lure C), so I will reject A”. In other words, the presence of more critical lures per list at the recognition test would increase the chance of feeling the contrast between a lure and the rest of the words (i.e., studied and other lures from the same list). Hence, just as in other manipulations that facilitate error-editing processes, overall false recognition rates were expected to be lower in the three-critical-lure condition than in the one-critical-lure condition.

Regarding the BAS manipulation, we used DRM lists from a previous normative study (Cadavid and Beato, 2017) where the low-BAS lists had the lowest possible BAS levels. In this case, as referred above, we expected that extremely low levels of associative strength would serve as a proxy for low-identifiability levels. That is, including extremely low-BAS lists allows us to virtually eliminate the possibility that participants engage in an identify-to-reject strategy.

Hence, as previously mentioned, the low-BAS/three-critical-lure condition would be the condition where it seems less likely that monitoring can occur via mutual exclusivity processes as (1) it is not likely that participants remember all the studied words (i.e., exhaustive recognition), (2) the lists had extremely low-BAS levels, which hinders the engagement of an identify-to-reject strategy, and (3) more critical lures at test increase the likelihood of experiencing feelings of contrast between the actually studied words and the critical lures.

We predicted that false recognition would be lower in the three-critical-lure condition than in the one-critical-lure condition, showing evidence toward the presence of feelings of contrast in the DRM paradigm. Furthermore, we were interested in analyzing two specific comparisons. First, we compared false recognition in the one-critical-lure vs. the three-critical-lure conditions for the low-BAS lists, as the extremely low-BAS levels prevent the use of an identify-to-reject strategy. We anticipated a lower false recognition rate in the low-BAS/three-critical-lure condition than in the low-BAS/one-critical-lure condition. This finding would show that, in the absence of an identify-to-reject strategy (low-BAS lists), participants whose feelings of contrast were not triggered (one-critical-lure condition) would show higher false memory than the participants who experienced feelings of contrast (three-critical-lure condition).

Our second specific comparison referred to BAS levels in the three-critical-lure condition. It is worth reminding that, in this study, BAS levels were used as a proxy for identifiability levels. We expected higher false recognition levels in the extremely low-BAS lists than in the high-BAS lists when tested with all its three critical lures. From our monitoring process perspective, as it was previously mentioned, in the experimental condition in which low-BAS lists are studied and three critical lures per list are included at test, participants could engage in a feeling-of-contrast strategy, but not in monitoring processes following the logic of mutual exclusivity (i.e., identify-to-reject strategy). However, when participants included in the three-critical-lure condition study high-BAS lists, it might be the case that they could use both feelings of contrast and an identify-to-reject strategy to monitor their memory (i.e., high-BAS lists could be used as a proxy for high-identifiability levels). Therefore, when using high-BAS lists and presenting three critical lures per list at test, participants could engage in more than one type of editing processes. If we assumed that there could be an additive effect on their ability to reduce false memories, this would lead to a greater reduction of false recognition in high-BAS lists than in low-BAS lists when presenting three critical lures per list at test.

## Materials and Methods

### Participants

A total of 140 undergraduate students, who were native Spanish speakers, voluntarily participated in this experiment (69.29% woman; *M*_*age*_ = 21.35, *SD* = 3.91). A power analysis using G^∗^Power 3.1 (Faul et al., 2007), with a power of 0.80 and an alpha of 0.05, showed that a total of 128 participants were enough to detect a medium effect size (*f* = 0.25) in the 2 (Number of critical lures per list at test) × 2 (BAS) interaction of our interest. We increased the sample size from 32 to 35 participants per group for a total of 140 participants. All participants signed an informed consent form and received course credit. The study was approved by the Bioethics Committee of the University of Salamanca.

### Design

The experiment followed a 2 × 2 between-subjects design. The two independent variables were Number of critical lures per list at test (one, three) and BAS (high, low).

### Materials

We used 32 six-word DRM lists from Cadavid and Beato (2017) normative study (see Table 1). Specifically, lists were constructed from Spanish free-association norms (Fernandez et al., 2011). Lists were built to ensure that all the three critical lures (e.g., WATER, BOAT, and SEA) were produced by the same study items (*marine, lifejacket, dyke, castaway, island*, and *exportation*) in free association. That is, the six study items were simultaneously related via backward associative strength or BAS to each of the three critical lures.

**TABLE 1:** Thirty-Two Six-Word Lists with Three Critical Lures (Approximated English Translation), Backward Associative Strength (BAS) Condition, and Critical Lure BAS.

**CRITICAL LURES: Associated words** **(Approximated English translation)**	**BAS condition**	**BAS Lure 1**	**BAS Lure 2**	**BAS Lure 3**
GUERRA, MALO, MIEDO: espía, infierno, puño, pelea, rapto, mortal (WAR, BAD, FEAR: spy, hell, fist, fight, abduction, mortal)	Low	0.013	0.010	0.010
EXAMEN, FÁCIL, TRABAJO: ejercicio, introducción, aplicación, exigencia, memoria, importante (EXAM, EASY, WORK: exercise, introduction, application, demand, memory, important)	Low	0.015	0.012	0.013
ANGUSTIA, LLORAR, PENA: llanto, afligido, deprimido, desazón, alivio, victimismo (ANGUISH, TO CRY, SORROW: crying, mournful, depressed, unease, relief, sense of victimization)	Low	0.012	0.012	0.017
HONOR, NOBLEZA, PERSONA: lealtad, nobiliario, integridad, orgullo, solemnidad, duque (HONOR, NOBILITY, PERSON: loyalty, nobility, integrity, pride, solemnity, duke)	Low	0.018	0.013	0.017
DOLOR, MUERTE, TRISTEZA: odio, hambre, inanición, morir, huérfano, consolado (PAIN, DEATH, SADNESS: hatred, hunger, starvation, to die, orphan, comforted)	Low	0.015	0.021	0.015
LIMPIEZA, SUCIEDAD, SUCIO: limpiar, gérmenes, basura, bastoncillo, fregadero, servilleta (CLEANLINESS, DIRT, DIRTY: to clean, germs, trash, cotton swab, sink, napkin)	Low	0.013	0.021	0.018
BOSQUE, CAMPO, MONTE: natural, conejo, valle, liebre, roble, refugio (FOREST, FIELD, HILL: natural, rabbit, valley, hare, oak, refuge)	Low	0.019	0.020	0.017
ALEGRÍA, CONTENTO, SONRISA: carcajada, jubiloso, animado, agrado, agradecer, esperanzado (JOY, PLEASED, SMILE: guffaw, jubilant, cheerful, kindness, to thank, hopeful)	Low	0.028	0.019	0.018
BEBÉ, CARIÑO, NIÑO: dulzura, hijo, tierno, protegido, acurrucarse, peluche (BABY, FONDNESS, CHILD: gentleness, son, tender, protected, to cuddle, teddy)	Low	0.018	0.025	0.027
CAMISA, PANTALÓN, ROPA: chaqueta, jersey, suéter, roto, rayas, arrugado (SHIRT, TROUSERS, CLOTHING: jacket, jersey, sweater, torn, stripes, wrinkled)	Low	0.024	0.024	0.028
DIVERSIÓN, FIESTA, NOCHE: club, marcha, droga, alcohol, concierto, cantar (FUN, PARTY, NIGHT: club, going out, drug, alcohol, concert, to sing)	Low	0.019	0.031	0.028
JUEZ, JUICIO, LEY: juramento, enmienda, justo, defensor, penal, defendido (JUDGE, TRIAL, LAW: oath, amendment, fair, defender, criminal, defendant)	Low	0.028	0.026	0.028
ENFERMO, HOSPITAL, MÉDICO: medicina, salud, dolencia, visita, virus, interno (SICK, HOSPITAL, DOCTOR: medicine, health, ailment, visit, virus, internal)	Low	0.028	0.023	0.033
INTELIGENCIA, LISTO, SABIO: erudición, genio, inculto, tenacidad, científico, elocuencia (INTELLIGENCE, SMART, WISE: erudition, genius, uncultured, tenacity, scientific, eloquence)	Low	0.023	0.032	0.032
CURA, IGLESIA, RELIGIÓN: papa, doctrina, blasfemia, reverencia, místico, súplica (CLERGYMAN, CHURCH, RELIGION: pope, doctrine, blasphemy, reverence, mystic, plea)	Low	0.035	0.027	0.030
CLASE, COLEGIO, ESCUELA: primaria, lección, aprender, academia, punzón, promoción (CLASS, SCHOOL, SCHOOL: elementary school, lesson, to learn, academy, punch, class)	Low	0.033	0.032	0.033
DESASTRE, HORROR, MUERTE: masacre, fatalidad, catástrofe, terremoto, tragedia, barbarie (DISASTER, HORROR, DEATH: massacre, fatality, catastrophe, earthquake, tragedy, brutality)	High	0.055	0.067	0.035
FOLIO, HOJA, PAPEL: doblar, margen, grapa, copia, clip, arrugado (FOLIO, SHEET, PAPER: to fold, margin, staple, copy, clip, crumpled)	High	0.053	0.045	0.062
DIOS, IGLESIA, MISA: mandamiento, oración, bendecir, devoción, comunión, gloria (GOD, CHURCH, MASS: commandment, prayer, to bless, devotion, communion, glory)	High	0.043	0.063	0.055
LÁGRIMA, LLORAR, TRISTEZA: lacrimal, llanto, despedida, emoción, infeliz, llover (TEAR, TO CRY, SADNESS: lachrymal, crying, farewell, emotion, unhappy, to rain)	High	0.061	0.055	0.068
ABRIGO, CHAQUETA, ROPA: cuero, gabardina, botón, colgar, chaleco, corchetes (COAT, JACKET, CLOTHING: leather, gabardine, button, to hang, vest, snap fastener)	High	0.066	0.074	0.049
ALCOHOL, BEBER, BEBIDA: ron, cerveza, tomar, botella, bar, copa (ALCOHOL, TO DRINK, DRINK: rum, beer, to drink, bottle, bar, drink)	High	0.066	0.065	0.072
BOSQUE, CAMPO, MONTE: excursión, seta, cabaña, ciervo, verde, pradera (FOREST, FIELD, HILL: excursion, mushroom, cottage, deer, green, meadow)	High	0.070	0.068	0.073
AMOR, CARIÑO, MADRE: ternura, dulzura, hijo, apreciación, consuelo, comprensión (LOVE, FONDNESS, MOTHER: tenderness, gentleness, son, fondness, comfort, comprehension)	High	0.092	0.069	0.065
CINE, PELÍCULA, TEATRO: actor, actriz, estrena, actuar, comedia, reparto (CINEMA, FILM, THEATER: actor, actress, premiere, to act, comedy, cast)	High	0.069	0.086	0.073
INTELIGENCIA, LISTO, SABIO: astucia, sabiduría, ingenio, erudición, genio, inculto (INTELLIGENCE, SMART, WISE: astuteness, wisdom, ingenuity, erudition, genius, uncultured)	High	0.077	0.080	0.075
CÁRCEL, LADRÓN, POLICÍA: detención, robo, mazmorra, delito, persecutoria, vigilancia (JAIL, THIEF, POLICE: detention, robbery, dungeon, crime, relative to persecution, vigilance)	High	0.088	0.060	0.089
FUMAR, HUMO, TABACO: pipa, puro, cenicero, pulmones, mechero, habano (TO SMOKE, SMOKE, TOBACCO: pipe, cigar, ashtray, lungs, lighter, havana cigar)	High	0.099	0.073	0.072
AGUA, BARCO, MAR: marina, salvavidas, dique, náufrago, isla, exportación (WATER, BOAT, SEA: marine, lifejacket, dyke, castaway, island, exportation)	High	0.072	0.088	0.090
MÚSICA, RUIDO, SONIDO: acústica, tambor, tono, cascabel, sonar, grillos (MUSIC, NOISE, SOUND: acoustic, drum, tone, rattle, to sound, crickets)	High	0.087	0.090	0.075
ENFERMEDAD, HOSPITAL, MÉDICO: clínica, sanidad, paciente, sarampión, dolencia, curar (DISEASE, HOSPITAL, DOCTOR: clinic, health service, patient, measles, ailment, to heal)	High	0.093	0.079	0.083
DINERO, SUELDO, TRABAJO: empleo, jornal, aumento, ganancias, jefe, mensual (MONEY, WAGE, WORK: job, day wage, raise, profits, boss, monthly)	High	0.085	0.078	0.104

*Lists Appear in Increasing Order of BAS per List.*

The BAS values for each critical lure (hereafter, *critical lure BAS*) were computed as the mean of the associative strengths between each of the six associated words and the particular critical lure, just as in previous research (Robinson and Roediger, 1997). For its part, the BAS values of each list (hereafter, *BAS list strength*) were calculated by averaging the BAS values for the three critical lures within each list. For example, a low-BAS list included the critical lures *WAR* (BAS = 0.013), *BAD* (BAS = 0.010), and *FEAR* (BAS = 0.010), each of which had backward associations to the study items *spy, hell, fist, fight, abduction*, and *mortal*. An example of a high-BAS list included the critical lures FOREST (BAS = 0.070), FIELD (BAS = 0.068), and HILL (BAS = 0.073), all of them associated with the study items *excursion, mushroom, cottage, deer, green*, and *meadow*.

Furthermore, ten DRM lists were selected from Alonso et al.’s (2004) normative study, from which the distractors were extracted. We used this normative study to select distractors from DRM lists that did not include, or were related to, our study items or critical lures. Lists included a critical lure (e.g., TELEPHONE) and fifteen associated words (e.g., *call, home, communication, mobile, dream, numbers, speak, invoice, conversation, guide, distance, cable, noise, chat*, and *prefix*). Specifically, we selected the lists of critical lures BOX, GLASSES, COMB, TRAVEL, KEY, FORK, LAMP, TELEPHONE, COW, and PENGUIN. Unrelated critical-distractors were the critical lure of the lists, whereas unrelated distractors were selected from its associates.

For the present study, we selected 16 high-BAS lists (*M*_*BAS*_ = 0.44, *SD* = 0.08, range_*per lure*_: 0.21–0.62) and 16 low-BAS lists (*M*_*BAS*_ = 0.13, *SD* = 0.04, range_*per lure*_: 0.06–0.21). It is important to note that the low-BAS lists included extremely low-BAS values, values never used before in the DRM paradigm, covering the lowest end of the entire spectrum of associative strength, as they included the minimum associative strength theoretically possible (Cadavid and Beato, 2017). We confirmed that the associative strengths of high- and low-BAS lists differed significantly, Welch’s *t*_*per lure*_(70.24) = 22.38, *p* < 0.001, *d* = 4.57. Lists were audio-recorded with a male voice, and during the study phase, stimuli were presented auditorily by using speakers.

The recognition test was administered as a pen and paper task. This memory test included a total of 192 words (studied words, critical lures, unrelated critical-distractors, and unrelated distractors) that varied between the experimental conditions. For the one-critical-lure condition, the recognition test consisted of 96 studied words, 16 critical lures (i.e., one critical lure per study list) and 80 distractors (10 unrelated critical-distractors and 70 unrelated distractors). We ensured that all the three critical lures of each list were tested in the one-critical-lure condition. For the three-critical-lure condition, the recognition test included 96 studied words, 48 critical lures (i.e., three critical lures per study list), and 48 distractors (8 unrelated critical-distractors and 40 unrelated distractors). Thus, the recognition memory test included the same number of studied and unstudied words (96 studied and 96 unstudied words) in both experimental conditions.

The items of the recognition test were pseudorandomized according to criteria proposed in previous research (Gallo and Roediger, 2002; Graham, 2007). Concretely, we made sure that two or more items separated words from the same list. Besides, we assured critical lures were separated from each other for at least two items. There were six versions of the recognition test, which was included at the end of a booklet that also contained sixteen pages of unsolved arithmetic operations series that were solved in-between the study of the lists.

### Procedure

Participants were randomly assigned to one of the four experimental conditions defined by two between-subjects variables: Number of critical lures per list at test (one, three) and BAS (high, low). This experiment was run in about 60-min group sessions. Before starting the study phase, participants were presented with a practice list. The study phase instructions indicated that words should be remembered for a later memory test. No mention was made about the associative nature of the study lists.

Participants studied 16 DRM lists randomly presented. The items within each list were presented in decreasing order of BAS values, at a rate of one word every 2000 ms. Lists were alternated with 20 s series of simple arithmetic operations that had to be solved in a booklet. The self-paced recognition test was administrated in the same booklet as the arithmetic operations. Participants had to judge 192 words and decide whether each word was presented in the study phase or not by circling “YES” or “NO” on the response sheet. As mentioned above, the number of critical lures included in the memory test varied between the one- and the three-critical-lure conditions (16 vs. 48, respectively).

## Results

Across all analyses, Greenhouse-Geisser correction of degrees of freedom was applied where appropriate in the repeated measures ANOVAs, the alpha level was set at 0.05, and effect sizes are reported with Cohen’s *d* and omega squared (ω^2^). All analyses were performed using JASP Team (2020).

### True Recognition

A 2 (Number of critical lures per list at test: one, three) × 2 (BAS: high, low) between-subjects ANOVA was ran on true recognition rates. No significant main effects, *F*_*number of lures*_(1, 136) = 0.04, *p* = 0.848, ω^2^ < 0.001, *F*_*BAS*_(1, 136) = 0.62, *p* = 0.432, ω^2^ < 0.001, nor interaction were found, *F*(1, 136) = 0.35, *p* = 0.557, ω^2^ < 0.001 (see Table 2 for descriptive statistics).

**TABLE 2:** Mean Percentage (SD) of True and False Recognition and False Alarms to Unrelated Critical-Distractors and Unrelated Distractors as a function of Number of Critical Lures per List at Test (one vs. three) and BAS (high vs. low).

	**One lure**		**Three lures**	
	**High BAS**	**Low BAS**	**High BAS**	**Low BAS**
True recognition	63.75 (14.83)	63.80 (9.86)	61.73 (12.04)	64.61 (12.73)
False recognition	41.25 (19.19)	42.14 (15.48)	26.19 (14.92)	36.90 (14.13)
False alarms to unrelated critical-distractors	8.86 (12.31)	11.18 (12.55)	6.79 (10.65)	12.14 (16.18)
False alarms to unrelated distractors	6.80 (6.36)	13.15 (11.26)	7.71 (10.58)	8.71 (9.22)

### False Recognition Effect

A one-way repeated measures ANOVA (Type of word: studied, critical lure, unrelated critical-distractor, and unrelated distractor) was conducted, *F*(2.42, 336.06) = 717.37, *p* < 0.001, ω^2^ = 0.74 (see Table 2 for descriptive statistics).^3^ We computed six comparisons and applied Bonferroni correction. Hence, the new alpha was set at 0.008. True recognition (*M* = 63.47, *SD* = 12.40) was significantly higher than false recognition to critical lures (*M* = 36.62, *SD* = 17.10), *t*(139) = 16.79, *p* < 0.001, Cohen’s *d* = 1.42; than false alarms to unrelated critical-distractors (*M* = 9.74, *SD* = 13.11), *t*(139) = 34.20, *p* < 0.001, Cohen’s *d* = 2.89; and also higher than false alarms to unrelated distractors (*M* = 9.09, *SD* = 9.75), *t*(139) = 39.58, *p* < 0.001, Cohen’s *d* = 3.35. Furthermore, false recognition was significantly higher than false alarms to unrelated critical-distractors, *t*(139) = 18.96, *p* < 0.001, Cohen’s *d* = 1.60; and unrelated distractors, *t*(139) = 21.69, *p* < 0.001, Cohen’s *d* = 1.83. This result confirmed that critical lures produced above-baseline levels of false recognition. No significant differences were found between unrelated critical-distractors and unrelated distractors, *t*(139) = 0.78, *p* = 0.435, Cohen’s *d* = 0.07.

### False Recognition and Critical Lures’ Position at Test

Besides using extremely low-BAS lists, the ease of engaging a feeling-of-contrast strategy during the recognition test was manipulated by including all the three critical lures of each list or including just one of them. According to the logic of the feeling of contrast, participants would experience more feelings of contrast when they encounter several critical lures. In order to check whether the feeling-of-contrast strategy was actually favored in the three-critical-lure condition, we analyzed false recognition of any of the three critical lures in the first, second or third position at test. Specifically, in the three-critical-lure condition, 35.24% (*SD* = 12.39) of the total false recognition occurred in the first critical lure of the lists, 35.91% (*SD* = 11.67) appeared in the second critical lure, and, finally, 28.84% (*SD* = 10.72) of the total false recognition happened in the third critical lure. We conducted a repeated measures ANOVA (Position at test of the critical lures of each list: first, second, and third) on the false recognition rates, *F*(2, 138) = 5.28, *p* = 0.007, ω^2^ = 0.06. We computed three comparisons and applied Bonferroni correction. Hence, the new alpha was set at 0.016. False recognition for the first and second critical lures did not show significant differences, *t*(69) = 0.26, *p* = 0.795, Cohen’s *d* = 0.03. However, false recognition for the third critical lure was significantly lower than false recognition for the first, *t*(69) = 2.68, *p* = 0.009, Cohen’s *d* = 0.32, and the second critical lure, *t*(69) = 3.17, *p* = 0.002, Cohen’s *d* = 0.38. These results supported the idea that the feeling-of-contrast strategy was favored in the three-critical-lure condition.

### Error-Editing Processes: The Effect of the Number of Critical Lures per List at Test and BAS

The mean percentage of false recognition as a function of the number of critical lures per list at test and BAS are presented in Table 2. In both one-critical-lure condition and three-critical-lure condition, false recognition was calculated as the mean of the false recognition of all the critical words included in the recognition test.

We used a 2 (Number of critical lures per list at test: one, three) × 2 (BAS: high, low) between-subjects ANOVA to examine the effects of the number of critical lures per list at test and BAS on the error-editing processes in false recognition. Results showed a significant main effect of BAS, *F*(1, 136) = 4.58, *p* = 0.034, ω^2^ = 0.02, indicating that false recognition rates were significantly higher in low-BAS lists (*M* = 39.52, *SD* = 14.95) than in high-BAS lists (*M* = 33.72, *SD* = 18.67). As expected, the number of critical lures per list at test also showed a significant main effect, *F*(1, 136) = 14.00, *p* < 0.001, ω^2^ = 0.09. Specifically, false recognition rates were lower in the three-critical-lure condition (*M* = 31.55, *SD* = 15.40) than in the one-critical-lure condition (*M* = 41.70, *SD* = 17.31). The interaction was not significant, *F*(1, 136) = 3.28, *p* = 0.072, ω^2^ = 0.02.

Since our goal was to analyze whether experiencing feelings of contrast could guide error-editing processes in DRM studies, we needed to eliminate the possibility that participants apply the mutual exclusivity logic (both via exhaustive recognition and identify-to-reject strategies). We assumed that the identify-to-reject strategy is mitigated with extremely low-BAS lists. However, no experimental manipulation was made to eliminate the possibility that participants engage in an exhaustive recognition strategy (i.e., recognize all the six study items). Therefore, we ran an additional conditioned analysis removing, for each participant, false recognition for lists that had perfect correct recognition for list items. This approach provided a better estimate of the feelings of contrast effects on false recognition, as we removed the lists in which mutual exclusivity could be occurring via exhaustive recognition. Participants in the low-BAS condition had 13.57% of lists removed (*M*_*one critical lure*_ = 12.68, *SD* = 13.93; *M*_*three critical lures*_ = 14.46, *SD* = 10.59), whereas 16.96% of the lists were eliminated in the high-BAS condition (*M*_*one critical lure*_ = 18.04, *SD* = 13.96; *M*_*three critical lures*_ = 15.89, *SD* = 12.62). Turning to the number of critical lures per list at test, in the one-critical-lure condition, 15.36% of the lists were removed, and 15.18% of the lists for the three-critical-lure condition were not included in the conditionalized analysis.

Just as in the previous ANOVA, in this conditioned analysis the main effect of BAS showed that false recognition was higher in low- (*M* = 38.42, *SD* = 14.76) than in high-BAS lists (*M* = 31.25, *SD* = 18.86), *F*(1, 136) = 6.65, *p* = 0.01, ω^2^ = 0.04. Again, the main effect of the number of critical lures per list at test was also significant, *F*(1, 136) = 8.09, *p* = 0.005, ω^2^ = 0.05, showing that false recognition was lower in the three-critical-lure condition (*M* = 30.88, *SD* = 15.95) than in the one-critical-lure condition (*M* = 38.80, *SD* = 17.70). The interaction was not significant, *F*(1, 136) = 1.73, *p* = 0.191, ω^2^ = 0.005 (see Table 3).

**TABLE 3:** Mean Percentage (SD) of True and False Recognition After Removing Lists with 100% of True Recognition, as a Function of Number of Critical Lures per List at Test (one vs. three) and BAS (high vs. low).

	**One lure**		**Three lures**	
	**High BAS**	**Low BAS**	**High BAS**	**Low BAS**
True recognition	56.14 (12.08)	58.99 (7.50)	55.23 (10.66)	59.34 (11.62)
False recognition	37.04 (19.91)	40.56 (15.28)	25.46 (16.01)	36.30 (14.13)

Taking into account our hypotheses stated in the introduction, we computed two planned comparisons on the conditioned false recognition rates (alpha was adjusted to 0.025). First, in the low-BAS condition, no differences were found between one and three critical lures, *t*(68) = 1.21, *p* = 0.12, Cohen’s *d* = 0.289. Considering the limitations of the null hypothesis significance testing (Dienes, 2011), Bayesian analyses were run (H0 = no differences between the means, H1 = differences between the means). A Bayesian independent samples *t*-test indicated that the H0 is 2.17 times more likely than the H1, which represent anecdotal evidence in favor of the H0. Second, in the three-critical-lure condition, false recognition was higher when participants could not resort to an identify-to-reject strategy (low-BAS lists) than when they could use such a strategy (high-BAS lists), *t*(68) = 3.00, *p* = 0.004, Cohen’s *d* = 0.718. A Bayesian independent samples *t*-test indicated that the H1 is 10.21 times more likely than the H0, which corresponds to moderate to strong evidence in favor of H1.

In sum, since false recognition was lower in the three-critical-lure condition than in the one-critical-lure condition, it seems that feelings of contrast could guide monitoring processes in the DRM paradigm. However, in the absence of an identify-to-reject strategy (low-BAS conditions), participants who experienced feelings of contrast (three-critical-lure condition) did not show lower levels of false memory than participants in the one-critical-lure condition. Instead, evidence was favorable to the existence of an additive effect of the two monitoring strategies examined in this study (i.e., identify-to-reject and feelings of contrast), as false recognition was significantly lower when both strategies were allowed (high-BAS/three-critical-lure condition).

## Discussion

The current study focused on disqualifying monitoring, a type of decision process that helps participants to reject false memories through the recollection of collateral information. The recollection of collateral information is achieved by the engagement of recall-to-reject strategies. Participants engage in recall-to-reject strategies employing one or two metacognitive processes: (1) applying the logic of mutual exclusivity, or (2) experiencing a feeling of contrast between studied items and unstudied critical lures (Gallo and Lampinen, 2016). As, to our knowledge, no previous studies have attempted to directly examine whether participants can apply a recall-to-reject strategy based on feelings of contrast in the DRM paradigm context, our goal was to fill this gap.

In the present study, we used a new experimental design where participants would find it difficult to use the logic of mutual exclusivity (neither exhaustive recall nor identify-to-reject strategy) to edit false memories, and instead, they could only experience feelings of contrast between words. Specifically, we employed DRM lists with three critical lures each of them (words not included in the study list) and extremely low levels of backward associative strength (the minimum possible association levels). In the recognition test, we manipulated the number of critical lures per list presented at test (only one vs. all the three critical lures per list).

On the one hand, regarding the number of critical lures per list, participants studied all the associates of the lists following the same instructions at the encoding phase. Importantly, at the recognition test, participants were presented with either only one or all the three critical lures of the lists. We expected that participants who were presented with three critical lures per list at test would increase their ability to edit false memories, as they would experience stronger feelings of contrast than the participants who were presented with only one critical lure per list at test. Participants in the three-critical-lure condition would be more prone to think something like: “This word (i.e., A, one of the critical lures) seems familiar to me, but I probably have this feeling just because another related word was actually presented (e.g., critical lure B or studied word C), so I will reject A.” This type of reasoning, repeated all across the recognition test, would lead to lower false recognition levels in the three-critical-lure condition than in the one-critical-lure condition. In other words, when exposed to three critical lures per list at test, participants would have fewer false memories. The results supported our hypothesis, meaning that, in the DRM context, disqualifying monitoring could be guided by the experience of feelings of contrast between different types of words.

On the other hand, we manipulated the level of BAS, including extremely low-BAS lists, that is, lists with the lowest possible BAS level. We anticipated that such extremely low-BAS levels would make it difficult for participants to guess the critical lures. In fact, previous DRM studies with three-critical-lure lists have found a significant correlation between BAS and identifiability of the critical lures (Beato and Cadavid, 2016). Therefore, we expected that the exceptionally low-BAS levels would also make it difficult to resort to recall-to-reject strategies guided by the logic of mutual exclusivity (e.g., identify-to-reject). The results supported our hypotheses, showing that lists with extremely low-BAS levels produced higher false recognition rates than lists with high-BAS levels. As expected, this difference was specially important in the three-critical-lure condition, where participants were better at avoiding false recognition. Indeed, participants committed fewer mistakes in the three-critical-lure condition, and, within this condition, they were particularly efficient at reducing false memory in the high-BAS condition. These results are in favor of the possibility that, when combined together, the two monitoring strategies explored in this study (i.e., identify to reject and feelings of contrast) could trigger an additive effect to reduce false memory (high-BAS/three-critical-lure condition). This outcome needs to be explored further in future research.

The current findings help us to gain knowledge of the mechanisms that underlie memory accuracy. Memory editing is a complex phenomenon that comprises multiple sub-processes and different strategies. In this research, while the encoding instructions at the study phase were not manipulated, we did manipulate the BAS level of the lists and the number of critical lures per list available at the recognition test. We found that, when presented with more critical lures per list at test, participants are better at avoiding false memories than when they are presented just with one critical lure per list at test (48 vs. 16 critical lures, respectively). Therefore, we provided evidence that the amount of competing information available at test is determinant to trigger different memory editing mechanisms. Our data are consistent with Gallo and collaborators’ classification of the decisional processes that guide memory distortion avoidance (e.g., Gallo, 2004; Gallo et al., 2006; Gallo and Lampinen, 2016; Moore et al., 2020) and leave the door open to new questions. One possible future line of continuing this research would come from manipulations both at encoding and at test. For example, would different types of instructions trigger the sort of attributional process associated with experiencing feelings of contrast? Would these feelings always decrease false memory? Could explicitly drawing participants’ attention to their subjective memory experience reduce false memory? One potential limitation of the current study is that participants were never directly queried regarding the monitoring of individual test items. Future studies with DRM lists with three critical lures could benefit greatly from adopting think-aloud protocols like those used by Lampinen et al. (2005). These and other questions remain to be explored in future research.

## Data Availability Statement

The raw data supporting the conclusions of this article will be made available by the authors, without undue reservation.

## Ethics Statement

This study involving human participants was reviewed and approaved by the Bioethics Committee of the University of Salamanca. The participants provided their written informed consent to participate in this study.

## Author Contributions

SC and MSB conceived and planned the experiment, analyzed data, and wrote the original draft of the manuscript. MS and PBA reviewed and edited the manuscript. All authors contributed to the article and approved the submitted version.

## Conflict of Interest

The authors declare that the research was conducted in the absence of any commercial or financial relationships that could be construed as a potential conflict of interest.

## Publisher’s Note

All claims expressed in this article are solely those of the authors and do not necessarily represent those of their affiliated organizations, or those of the publisher, the editors and the reviewers. Any product that may be evaluated in this article, or claim that may be made by its manufacturer, is not guaranteed or endorsed by the publisher.

## Footnotes

1 Other theoretical explanations of false memories, such as global matching models (Arndt and Hirshman, 1998), also agree on proposing these two types of processes.

2 The backward associative strength or BAS has been identified as a reliable predictor of false memory and is one of the most commonly cited factors that facilitates error-inflating processes, as research has shown that false recall and false recognition rates were higher in high-BAS than in low-BAS lists (e.g., McEvoy et al., 1999; Roediger et al., 2001b; Gallo and Roediger, 2003; Arndt and Gould, 2006; Beato and Arndt, 2017). However, some studies have also reported that BAS did not affect false recognition rates (e.g., Cadavid et al., 2012; Brainerd et al., 2020). Although these results are of interest, the aim of the present study is not to shed light on the role of BAS in false memory, as the extremely low-BAS levels employed here are only used as a proxy for low-identifiability levels.

3 No correction was made on recognition rates because false alarms to unrelated critical-distractors did not show significant differences between the two experimental conditions, one critical lure (*M* = 10.02, *SD* = 12.40) and three critical lures per list at test (*M* = 9.46, *SD* = 13.86), *t*(138) = 0.25, *p* = 0.804, Cohen’s *d* = 0.04. Furthermore, false alarms to unrelated distractors did not show significant differences between the two experimental conditions either, *t*(138) = 1.07, *p* = 0.287, Cohen’s *d* = 0.18 (one-critical-lure condition, *M* = 9.97, *SD* = 9.62; three-critical-lure condition, *M* = 8.21, *SD* = 9.87).

## References

[B1] Alonso M. A. Fernandez A. Díez E. Beato M. S. (2004). Índices de producción de falso recuerdo y falso reconocimiento para 55 listas de palabras en castellano [False recall and recognition indexes for 55 lists of words in Spanish]. *Psicothema* 16 357–362.

[B2] Arndt J. Beato M. S. (2017). The role of language proficiency in producing false memories. *J. Memory Lang.* 95 146–158. 10.1016/j.jml.2017.03.004

[B3] Arndt J. Gould C. (2006). An examination of two-process theories of false recognition. *Memory* 14 814–833. 10.1080/09658210600680749 16938694

[B4] Arndt J. Hirshman E. (1998). True and false recognition in MINERVA2: Explanations from a global matching perspective. *J. Memory Lang.* 39 371–391. 10.1006/jmla.1998.2581

[B5] Beato M. S. Arndt J. (2014). False recognition production indexes in forward associative strength (FAS) lists with three critical words. *Psicothema* 26 457–463. 10.7334/psicothema2014.79 25340891

[B6] Beato M. S. Arndt J. (2017). The role of backward associative strength in false recognition of DRM lists with multiple critical words. *Psicothema* 29 358–363. 10.7334/psicothema2016.248 28693707

[B7] Beato M. S. Arndt J. (2021). The effect of language proficiency and associative strength on false memory. *Psycholog. Res.* 2021:1449. 10.1007/s00426-020-01449-3 33387022

[B8] Beato M. S. Boldini A. Cadavid S. (2012). False memory and level of processing effect: An event-related potential study. *NeuroReport* 23 804–808. 10.1097/WNR.0b013e32835734de 22811058

[B9] Beato M. S. Cadavid S. (2016). Normative study of theme identifiability: Instructions with and without explanation of the false memory effect. *Behav. Res. Methods* 48 1252–1265. 10.3758/s13428-015-0652-6 26424441

[B10] Beato M. S. Cadavid S. Pulido R. F. Pinho M. S. (2013). No effect of stress on false recognition. *Psicothema* 25 25–30. 10.7334/psicothema2012.158 23336539

[B11] Beato M. S. Díez E. (2011). False recognition production indexes in Spanish for 60 DRM lists with three critical words. *Behav. Res. Methods* 43 499–507. 10.3758/s13428-010-0045-9 21298572

[B12] Boldini A. Beato M. S. Cadavid S. (2013). Modality-match effect in false recognition: An event-related potential study. *NeuroReport* 24 108–113. 10.1097/WNR.0b013e32835c93e3 23370492

[B13] Brainerd C. J. Chang M. Bialer D. M. (2020). From association to gist. *J. Exp. Psychol. Learn. Memory Cogn.* 46 2106–2127. 10.1037/xlm0000938 32658546

[B14] Brainerd C. J. Reyna V. F. (1990). Gist is the grist: Fuzzy-trace theory and the new intuitionism. *Dev. Rev.* 10 3–47. 10.1016/0273-2297(90)90003-M

[B15] Brainerd C. J. Reyna V. F. Ceci S. J. (2008). Developmental reversals in false memory: a review of data and theory. *Psycholog. Bull.* 134 343–382. 10.1037/0033-2909.134.3.343 18444700

[B16] Brainerd C. J. Reyna V. F. Wright R. Mojardin A. H. (2003). Recollection rejection: false-memory editing in children and adults. *Psycholog. Rev.* 110 762–784. 10.1037/0033-295X.110.4.762 14599242

[B17] Cadavid S. Beato M. S. (2016). Memory distortion and its avoidance: An event-related potentials study on false recognition and correct rejection. *PLoS One* 11:e0164024. 10.1371/journal.pone.0164024 27711125 PMC5053520

[B18] Cadavid S. Beato M. S. (2017). False recognition in DRM lists with extremely low association: A normative study. *Psicologica* 38 133–147.

[B19] Cadavid S. Beato M. S. Fernandez A. (2012). Falso reconocimiento en listas DRM con tres palabras críticas: Asociación directa vs. inversa [False recognition in DRM lists with three critical words: Forward vs. backward association]. *Psicologica* 33 39–58.

[B20] Carneiro P. Fernandez A. Dias A. R. (2009). The influence of theme identifiability on false memories?: Evidence for age-dependent opposite effects. *Memory Cogn.* 37 115–129. 10.3758/MC.37.2.115 19223562

[B21] Carneiro P. Fernandez A. Díez E. Garcia-Marques L. Ramos T. Ferreira M. B. (2012). “Identify-to-reject”: A specific strategy to avoid false memories in the DRM paradigm. *Memory Cogn.* 40 252–265. 10.3758/s13421-011-0152-6 21971606

[B22] Coane J. H. McBride D. M. Xu S. (2020). The feature boost in false memory: The roles of monitoring and critical item identifiability. *Memory* 28 481–493. 10.1080/09658211.2020.1735445 32107971

[B23] Deese J. (1959). On the prediction of occurrence of particular verbal intrusions in immediate recall. *J. Exp. Psychol.* 58 17–22. 10.1037/h0046671 13664879

[B24] Dienes Z. (2011). Bayesian versus orthodox statistics: Which side are you on? *Perspect. Psycholog. Sci.* 6 274–290. 10.1177/1745691611406920 26168518

[B25] Faul F. Erdfelder E. Lang A. G. Buchner A. (2007). G^∗^Power 3: A flexible statistical power analysis program for the social, behavioral, and biomedical sciences. *Behav. Res. Methods* 39 175–191. 10.3758/BF03193146 17695343

[B26] Fernandez A. Díez E. Alonso M. A. (2011). *Materiales normativos en castellano: Normas de asociación libre [Normative materials in Castilian: Free association norms].* Available online at: http://campus.usal.es/gimc/nalc

[B27] Gallo D. A. (2004). Using recall to reduce false recognition: Diagnostic and disqualifying monitoring. *J. Exp. Psychol. Learn. Memory Cogn.* 30 120–128. 10.1037/0278-7393.30.1.120 14736301

[B28] Gallo D. A. (2006). *Associative illusions of memory: False memory research in DRM and related tasks.* London: Psychology Press.

[B29] Gallo D. A. (2010). False memories and fantastic beliefs: 15 years of the DRM illusion. *Memory Cogn.* 38 833–848. 10.3758/MC.38.7.833 20921097

[B30] Gallo D. A. Bell D. M. Beier J. S. Schacter D. L. (2006). Two types of recollection-based monitoring in younger and older adults: Recall-to-reject and the distinctiveness heuristic. *Memory* 14 730–741. 10.1080/09658210600648506 16829489

[B31] Gallo D. A. Lampinen J. M. (2016). “Three pillars of false memory prevention: Orientation, evaluation, and corroboration,” in *The Oxford handbook of metamemory*, Chap. Oxford, eds Dunlosky J. Uma S. Tauber K. (Oxford: Oxford University Press), 387–403. 10.1093/oxfordhb/9780199336746.013.11

[B32] Gallo D. A. Roediger H. L. (2002). Variability among word lists in eliciting memory illusions: Evidence for associative activation and monitoring. *J. Memory Lang.* 47 469–497. 10.1016/S0749-596X(02)00013-X

[B33] Gallo D. A. Roediger H. L. (2003). The effects of associations and aging on illusory recollection. *J. Memory Lang.* 47 469–497. 10.3758/BF03196124 14704018

[B34] Graham L. M. (2007). Need for cognition and false memory in the Deese–Roediger–McDermott paradigm. *Personal. Individ. Diff.* 42 409–418. 10.1016/j.paid.2006.07.012

[B35] Gray S. G. (2016). *Transcranial direct current stimulation and episodic memory retrieval* [Doctoral dissertation, Department of Psychology. Chicago: University of Chicago, 10.6082/M13F4MHX

[B36] Howe M. L. Akhtar S. (2020). Priming older adults and people with mild to moderate Alzheimer’s disease problem-solving with false memories. *Cortex* 125 318–331. 10.1016/j.cortex.2020.01.014 32113046

[B37] Huff M. J. Bodner G. E. Gretz M. R. (2020). Reducing false recognition in the Deese-Roediger/McDermott paradigm: Related lures reveal how distinctive encoding improves encoding and monitoring processes. *Front. Psychol.* 11:602347. 10.3389/fpsyg.2020.602347 33329270 PMC7714777

[B38] Huff M. J. Bodner G. E. Gretz M. R. (2021). Distinctive encoding of a subset of DRM lists yields not only benefits, but also costs and spillovers. *Psycholog. Res.* 85 280–290. 10.1007/s00426-019-01241-y 31463566

[B39] JASP Team (2020). *JASP* (Version 0.14.1) [Computer software].

[B40] Jou J. Escamilla E. E. Torres A. U. Ortiz A. Perez M. Zuniga R. (2018). Why seemingly more difficult test conditions produce more accurate recognition of semantic prototype words: A recognition memory paradox? *Conscious. Cogn.* 63 239–253. 10.1016/j.concog.2018.06.003 30008339

[B41] Lampinen J. M. Meier C. R. Arnal J. D. Leding J. K. (2005). Compelling untruths: Content borrowing and vivid false memories. *J. Exp. Psychol. Learn. Memory Cogn.* 31 954–963. 10.1037/0278-7393.31.5.954 16248744

[B42] Liu H. Gao Q. Zheng L. Wu Y. Wang C. Weng X. (2020). The neural correlates of context retrieval in false recognition. *NeuroReport* 31 966–970. 10.1097/WNR.0000000000001502 32639271

[B43] Liu Z. Liu T. Li Y. (2021). How does social competition affect true and false recognition? *Psychon. Bull. Rev.* 28 292–303. 10.3758/s13423-020-01807-7 32935280

[B44] McEvoy C. L. Nelson D. L. Komatsu T. (1999). What is the connection between true and false memories? The differential roles of interitem associations in recall and recognition. *J. Exp. Psychol.* 25 1177–1194. 10.1037/0278-7393.25.5.1177 10505342

[B45] Moore K. N. Lampinen J. M. Bridges A. J. Gallo D. A. (2020). Developmental trends in children’s use of different monitoring processes to avoid false memories. *Cogn. Dev.* 55:100911. 10.1016/j.cogdev.2020.100911

[B46] Nieznański M. Obidziński M. Niedziałkowska D. Zyskowska E. (2018). Context recollection and false memory of critical lures in the Deese/Roediger-McDermott paradigm: The role of encoding-and retrieval-based mechanisms. *Psihologijske Teme* 27 365–384. 10.31820/pt.27.3.2

[B47] Pitarque A. Satorres E. Sales A. Escudero J. Meléndez J. C. (2018). Effects of stimuli repetition and age in false recognition. *Psycholog. Rep.* 121 1106–1119. 10.1177/0033294117747284 29298586

[B48] Reyna V. F. Brainerd C. J. (1995). Fuzzy-trace theory: An interim synthesis. *Learn. Indiv. Diff.* 7 1–75. 10.1016/1041-6080(95)90031-4

[B49] Robinson K. J. Roediger H. L. (1997). Associative processes in false recall and false recognition. *Psycholog. Sci.* 8 231–237. 10.1111/j.1467-9280.1997.tb00417.x

[B50] Roediger H. L. Balota D. A. Watson J. M. (2001a). “Spreading activation and arousal of false memories,” in *Science conference series. The nature of remembering: Essays in honor of Robert G. Crowder*, eds Roediger H. L. Nairne J. S. Neath I. Surprenant A. M. (Washington, D.C: American Psychological Association), 95–115. 10.1037/10394-006

[B51] Roediger H. L. McDermott K. B. (1995). Creating false memories: Remembering words not presented in lists. *J. Exp. Psychol.* 21 803–814. 10.1037/0278-7393.21.4.803

[B52] Roediger H. L. Watson J. M. McDermott K. B. Gallo D. A. (2001b). Factors that determine false recall: A multiple regression analysis. *Psychon. Bull. Rev.* 8 385–407. 10.3758/BF03196177 11700893

[B53] Thakral P. P. Madore K. P. Devitt A. L. Schacter D. L. (2019). Adaptive constructive processes: An episodic specificity induction impacts false recall in the Deese-Roediger- McDermott paradigm. *J. Exp. Psychol. Gen.* 148 1480–1493. 10.1037/xge0000577 30829522 PMC6715504

[B54] Wang J. Otgaar H. Santtila P. Shen X. Zhou C. (2021). How culture shapes constructive false memory. *J. Appl. Res. Memory Cogn.* 2021:002. 10.1016/j.jarmac.2020.12.002.12.002

